# Perspectives and Challenges of Telemedicine and Artificial Intelligence in Pediatric Dermatology

**DOI:** 10.3390/children11111401

**Published:** 2024-11-19

**Authors:** Daniele Zama, Andrea Borghesi, Alice Ranieri, Elisa Manieri, Luca Pierantoni, Laura Andreozzi, Arianna Dondi, Iria Neri, Marcello Lanari, Roberta Calegari

**Affiliations:** 1 Department of Medical and Surgical Sciences, Alma Mater Studiorum, University of Bologna, 40138 Bologna, Italy; daniele.zama2@unibo.it (D.Z.); iria.neri@aosp.bo.it (I.N.); marcello.lanari@unibo.it (M.L.); 2 Pediatric Emergency Unit, IRCCS Azienda Ospedaliero-Universitaria di Bologna, 40138 Bologna, Italy; luca.pierantoni@aosp.bo.it (L.P.); laura.andreozzi4@unibo.it (L.A.); 3 Department of Computer Science and Engineering (DISI), Alma Mater Studiorum, University of Bologna, 40126 Bologna, Italy; andrea.borghesi3@unibo.it (A.B.); roberta.calegari@unibo.it (R.C.); 4 Specialty School of Paediatrics, Alma Mater Studiorum, University of Bologna, 40126 Bologna, Italy; alice.ranieri3@studio.unibo.it (A.R.); elisa.manieri3@studio.unibo.it (E.M.); 5 Dermatology Unit—IRCCS, Azienda Ospedaliero-Universitaria, Policlinico Sant’Orsola-Malpighi, 40138 Bologna, Italy

**Keywords:** artificial intelligence, children, dermatologists, pediatric dermatology, telemedicine

## Abstract

Background: Pediatric dermatology represents one of the most underserved subspecialties in pediatrics. Artificial intelligence (AI) and telemedicine have become considerable in dermatology, reaching diagnostic accuracy comparable to or exceeding that of in-person visits. This work aims to review the current state of telemedicine and AI in pediatric dermatology, suggesting potential ways to address existing issues and challenges. Methods: We conducted a literature review including only articles published in the last 15 years. A total of 458 studies were identified, of which only 76 were included. Results: Most of the studies on telemedicine evaluate accuracy focused on concordance, which ranges from 70% to 89% for the most common pediatric skin diseases. Telemedicine showed the potential to manage chronic dermatological conditions in children, as well as decrease waiting times, and represents the chance for unprivileged populations to overcome barriers limiting access to medical care. The main limitations of telemedicine consist of the language barrier and the need for adequate technologies and acceptable image-quality video, which can be overcome by AI. AI-driven apps and platforms can facilitate remote consultations between pediatric dermatologists and patients or their caregivers. However, the integration of AI into clinical practice faces some challenges ranging from technical to ethical and regulatory. It is crucial to ensure that the development, deployment, and utilization of AI systems conform to the seven fundamental requirements for trustworthy AI. Conclusion: This study supplies a detailed discussion of open challenges with a particular focus on equity and ethical considerations and defining possible concrete directions.

## 1. Introduction

The sharing of healthcare data and the spread of digital health tools present an opportunity to overcome the challenges posed by geographic, sociocultural, and economic barriers and the shortage of professionals in specific medicine subspecialties. Pediatric dermatology represents a paradigmatic example where telemedicine and artificial intelligence (AI) may play a pivotal role. The link between telemedicine and AI lies in the latter’s ability to enhance the former by enhancing its quality, accuracy, and diagnostic application. Moreover, pediatric dermatology constitutes one of the most underserved subspecialties in childhood medicine [1]. Skin-related problems involve between 10% and 30% of pediatric primary care visits, leading to a high request for consultation. There is a pronounced shortage of pediatric dermatologists and general dermatologists adequately trained in this field [2].

After the COVID-19 pandemic, teledermatology was proposed as a way to expand dermatological services for pediatric patients [3,4], reduce in-person contact, and continue supplying healthcare [5,6,7]. Moreover, AI is being developed to diagnose and manage skin conditions in children. AI and telemedicine are gaining growing importance in dermatology, with studies indicating diagnostic accuracy comparable to or exceeding that of dermatologists for skin lesions diagnosed from clinical and dermoscopic images [8]. Indeed, studies on pediatric cases are limited, and real-world clinical validation is currently lacking.

The significance of dedicated pediatric approaches stems from the recognition that children present different and sometimes unique pathologies or distinct manifestations compared to adults. There may also be potential genetic forms emerging in childhood. Additionally, there are challenges in patient collaboration mainly during preschool age, and the involvement of parents or caregivers in the interactions with healthcare providers. Some factors, including therapeutic compliance and adherence to follow-ups, are closely tied to the parent or caregiver. Therefore, how pediatric patients engage with telemedicine and AI differs from that of adults.

The present work aims to review the current status of telemedicine and AI in pediatric dermatology and analyze their current limitations and practical problems. In addition, this paper suggests potential ways to address the issues and challenges through the development of frameworks, supporting the development of an AI system in the field of pediatric dermatology as one applied in our hospital, the Sant’Orsola University Hospital in Bologna, as described below (Assessment and Engineering of Equitable, Unbiased, Impartial, and Trustworthy AI Systems).

## 2. Materials and Methods

**Figure 1:**
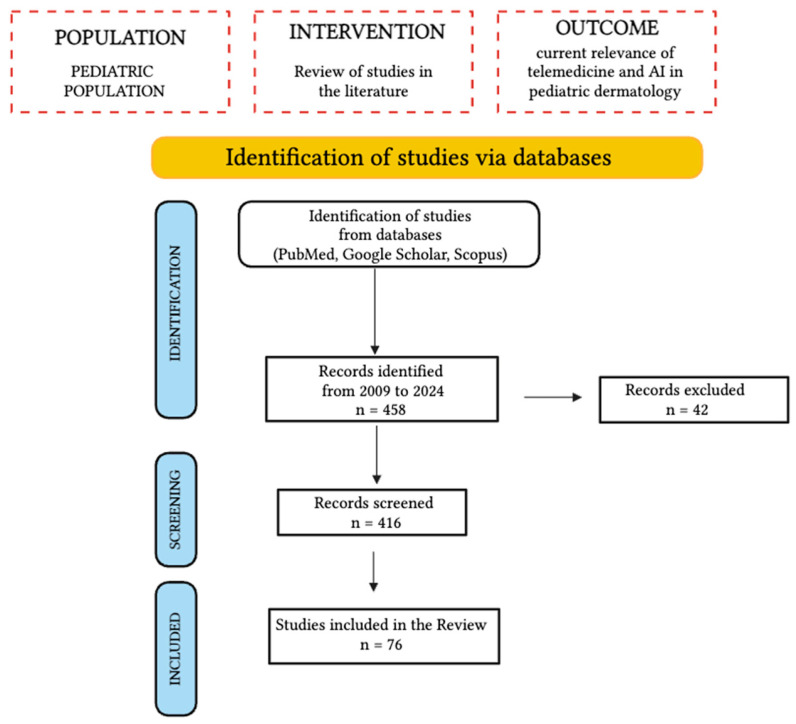
A PRISMA flowchart of the study.

The present review aims to examine the current status of telemedicine and AI in pediatric dermatology, analyze their current limitations, and suggest potential ways to address existing issues and challenges. For this reason, we conducted a literature review of studies concerning telemedicine and AI in pediatric dermatology. Pubmed, Google Scholar, and Scopus were the three databases screened, based on a combination of the following terms: “telemedicine” AND “pediatric dermatology”, “pediatric” AND “teledermatology”, and “artificial intelligence” AND “pediatric dermatology”. Only articles published in English and in the last 15 years were included. Studies published until July 2024 were included. Regarding the search on Google Scholar, only the first five pages of each search were checked. A total of 458 studies were identified, of which, after removing non-related studies and overlaps, only 76 were included. Papers on teledermatology or AI in dermatology that specifically focused on an adult population were excluded, as were articles dealing solely with telemedicine and AI in adult dermatology. The PRISMA flowchart method is used to outline the aim of the study (Figure 1).

We analyzed AI applications in pediatric dermatology and discussed their current capabilities, potential failure modes, and challenges surrounding performance assessment and interpretability (and more generally, reliability). The following primary applications were addressed: (i) teledermatology, including triage for referral to dermatologists, and (ii) dermatopathology. This paper discusses equity and ethical issues related to future clinical adoption and recommends possible directions to generate trust in this process.

## 3. Results

The results of our research are summarized below, dividing those relating to telemedicine from those relating to AI.

### 3.1. Telemedicine Applications in Pediatric Dermatology Subsection

Teledermatology refers to the method of delivering dermatological care services from a distance using telecommunication technology [2]. There are three main models of teledermatology: synchronous, asynchronous, and hybrid or mixed [9]. Synchronous teledermatology involves real-time video interaction between the primary care providers (PCPs), the patient, and the teledermatologist [2]. An asynchronous modality uses store-and-forward (SAF) technology, using an electronic platform where images are obtained by the requesting clinician, the patient, and/or family and sent to the responding dermatologist for consultation [3,10]. Hybrid or mixed models are a combination of both modalities.

Diagnostic accuracy and concordance (or reliability) are measures to compare a virtual pediatric consultation to a diagnosis from an in-person visit. Diagnostic accuracy is evaluated by comparing the diagnosis determined via teledermatology with a gold standard procedure (i.e., histopathology) [11]. Diagnostic concordance or reliability refers to the interobserver agreement between a teledermatology diagnosis and an in-person visit. Most of the studies evaluating teledermatology accuracy focus on concordance [10,11]. Diagnostic concordance is reported to be comparatively high, ranging from 70% to 89% for the most common pediatric skin diseases (rashes, birthmarks, inflammatory dermatoses, infections, nodules, and alopecia-related diagnosis) [12,13,14,15,16]. The highest discordance was found for conditions frequently reported in the pediatric field, such as pityriasis rosea, tinea versicolor, seborrheic dermatitis, xerosis, and lichen striatus. Chen et al. conducted a retrospective study on a cohort of 429 patients under the age of 13 and found that 42% of cases had inconsistencies in their diagnosis [17]. Chen’s study reveals a considerable management discordance (36%) between the referring physician and the teledermatologist, indicating the underuse of medications, i.e., topical steroids. The most common pediatric diagnoses evaluated in teledermatology include acne, molluscum contagiosum, verruca vulgaris (warts), atopic dermatitis, and benign melanocytic nevi [2,5,12,13,18]. In a retrospective and observational study led by Batalla et al. in 2015, the three main groups of diagnosed conditions were inflammatory diseases, benign pigmented lesions, and infectious diseases [13]. It was also reported that almost half of the consultations requested face-to-face detection for “diagnostic confirmation.”

Several papers have investigated the diagnostic concordance between virtual and in-person dermatological assessments in pediatric patients. A groundbreaking study, conducted by O’Connor et al. in 2017, utilized a prospective design involving 40 pediatric patients [14]. The study demonstrated a high level of accuracy in virtual dermatological assessments, with a diagnostic concordance rate of 83% compared to in-person consultations, emphasizing the potential role of telemedicine as a reliable tool in pediatric dermatology. O’Connor’s study systematically addressed the agreement between diagnoses based on parental photographs associated with pediatric teledermatology and those based on face-to-face examinations. The research highlighted strategies to overcome limitations in visual assessments in children, such as the use of parent-assisted imaging. Taslidere et al. (2023) reported an overall concordance rate of 74.2%, indicating substantial agreement between virtual and face-to-face diagnoses [19]. Factors influencing diagnostic concordance, such as image quality and the use of advanced technologies, were explored. Tollefson et al. (2012) followed a cohort of pediatric patients with infantile hemangioma (IH)8 through virtual consultations, showing consistent and reliable monitoring over 6 months [20]. These findings underscore the potential of telemedicine for managing chronic dermatological conditions in children. Moreover, Betlloch-Mas et al. examined 131 IH patients who were treated between 2008 and 2018. The study demonstrated that the use of telemedicine resulted in a faster commencement of treatment with propranolol, which led to a decrease in the age of treatment initiation [21]. During the COVID-19 pandemic, the Hemangioma Investigator Group (HIG) released consensus recommendations for the management of IH via telemedicine. These recommendations were categorized into standard and high-risk forms. According to the HIG, telehealth could be a good alternative for the handling and treatment of the first group [22]. In a cross-sectional study, Kittler et al. found that the median physician confidence in managing and evaluating IH through telemedicine was 95.0 on a scale ranging from 0 to 100 [23]. Teledermoscopy refers to the transmission of a dermoscopy image by the PCP, which is shared with the dermatologist via online systems. Some authors investigated the usefulness of PCPs sharing dermoscopic pictures with dermatologists via online systems (teledermoscopy) to improve diagnostic accuracy. It appeared to be effective in diagnosing pigmented lesions and also as a triage tool with an in-person follow-up visit [10,24,25]. The management agreement is defined by the PCP and dermatologist on a given method of treatment, and it spans from 25% to 44% [12]. A study by Ying et al. [26] investigating PCP’s management of eczema highlighted how telemedicine can be helpful but insufficient to cope with the acquisition of pediatric eczema guidelines by primary caregivers. Moreover, a survey in the UK suggested that teleconsulting has a more central role in follow-up rather than as new visits [27].

One of the potential main benefits linked to the increased use of telemedicine, which emerged during the COVID-19 pandemic, is its cost-efficiency [28]. In the field of pediatric teledermatology, several studies reported a decrease in the need for in-person follow-up after a telemedicine appointment. Specifically, there has been a reduction of almost 23% in the number of in-person visits compared to the total number of visits since the onset of the COVID-19 pandemic [13,15,21,29,30,31].

Since the COVID-19 pandemic, the main conditions for in-person visits and management were pediatrician demand, diagnostic doubt, procedure/therapeutic intervention, and absence of response to treatment [13,32]. The main indications were alopecia, pigmented lesions, and warts. Furthermore, in a retrospective study, children who received a recommendation for face-to-face evaluation were younger on average than those who did not [29].

Telemedicine may also potentially serve as a tool to decrease waiting times, which represent a consistent obstacle in this field [33,34]. A survey conducted by Prindavile et al. in 2018 found that the average time for a pediatric dermatological appointment in the US is 6 weeks [34]. In 2020, Seiger et al. [29] reported a 31% reduction in appointment waiting times when teledermatology was utilized. In a study by Kittler et al. [23], the median time from forwarding to assessment was 17 days. A 2021 retrospective cross-sectional study conducted in Germany [35] demonstrated a substantial decrease in waiting times due to teledermatology, going from an average time of 4.9 weeks for an in-person dermatologist appointment to 90% of the cases being addressed on the same or the following day.

Teledermatology also offers a potential advantage for unprivileged populations by helping them overcome barriers that prevent them from accessing medical care (e.g., the lack of support with childcare, financial constraints, limited transportation, and communication) [4,33]. A multicenter study described the impact of telemedicine on the no-show rate in a Federally Qualified Health Center situated in Brooklyn, NY, USA, which delivers medical care to minority populations, the uninsured, and patients below the poverty line. The study compared two different 7-month periods, where only in-person visits were performed in the first period (1189 total scheduled visits) and both virtual and in-person visits in the second (1064 scheduled visits), and showed how the introduction of telehealth reduced the number of missed pediatric appointments by almost 40% [36,37].

Although a significant increase in the use of telemedicine has been reported among dermatologists, pediatric patients, and parents [29,38,39], certain factors leading to dissatisfaction emerged. These factors highlight the potential limitations that need to be addressed in the future. The challenges consist of difficulties in collecting a complete pediatric medical history (including birth and developmental history) that can save time, the impossibility of a complete examination, the lack of direct contact, and varying levels of digital proficiency [2,18]. In this regard, a prospective study analyzed a UK single-center cohort of pediatric dermatology patients managed via telephone consultations during the COVID-19 pandemic. The study collected data on outcomes from clinicians and included a qualitative questionnaire filled out by patients and parents. It highlighted the divergence between the practical success of teledermatology from the physician’s viewpoint and the parental perspective. Among the parents surveyed, 52% expressed dissatisfaction with telephone management, and most of them (64%) stated that they preferred face-to-face visits in the future. This highlights the tendency for parents to have less confidence in virtual treatment for their children and instead prefer a physical examination [18] and suggests the need for a teleconsultation model suited to the patient and the physician [27].

Some groundbreaking care models can implement and improve the use of telemedicine. The Extension for Community Healthcare Outcomes (ECHO) is a project that was first developed in New Mexico, USA, to strengthen the abilities of PCPs across different medical areas [40,41]. It consists of multidisciplinary teleconferencing, which connects specialists with PCPs in remote or rural areas using a telemonitoring program [40,41]. A retrospective study valued the application and impact of the ECHO in 137 adults and 44 pediatric dermatology cases over 2 years, showing how this project constitutes a teaching modality that increases PCPs’ abilities to treat dermatologic conditions [40].

The original studies on the application of telemedicine in pediatric dermatology are reported in Table 1.

### 3.2. The Role of Artificial Intelligence in Pediatric Dermatology

The term AI refers to the development of computer systems able to perform tasks that would normally require human intelligence. There are various types of AI: assisted, which executes defined tasks; augmented, which may improve human intelligence and competencies; and autonomous intelligence, where systems can generate intelligence autonomously [46]. Machine learning (ML) is a field of artificial intelligence that enables machines to automatically learn from data and past experiences to identify patterns and make predictions with minimal human intervention. ML can be roughly classified as supervised or unsupervised, according to the availability of annotated data. Recent advances in ML fall under the category of deep learning (DL), where the models have become more complex; the latter is the most widely used model in healthcare [47]. DL is dependent on artificial neural networks (ANNs), which consist of layered sets of trainable weights that mimic the human brain’s capability to recognize patterns within data (by adjusting the intensity of the connections between neighboring neurons). Therefore, DL systems have the capability of adjusting their weights independently of human programming [48].

AI has the potential to make a significant contribution to pediatric dermatology by assisting healthcare providers in diagnosing common skin conditions or in therapeutic decision-making, while also providing parents with faster initial assessments. As teledermatology presents some limitations, such as the language barrier and the need for adequate technologies and acceptable image-quality video, AI may overcome these disparities by linking clinical history and photo-reviewed ML [49]. This combination may help in the diagnosis and management of skin disorders by non-specialists, therefore compensating for the lack of access to a pediatric dermatologist, which is often difficult to obtain [49].

To date, the use of AI applications in dermatology has mainly been confined to the adult population, and studies on pediatric cohorts have been claimed [49].

AI-driven apps and platforms can facilitate remote consultations between pediatric dermatologists and patients or their caregivers, serving as a point of care in underserved areas and providing preliminary assessments.

In the context of pediatric dermatology, the study by Zhang et al. was the first to show how AI can be applied. The authors collected 79,675 clinical images from pediatric dermatology patients at the Children’s Wisconsin Hospital, USA, taken by a group of families, physicians, nurses, and medical assistants over the span of 15 years in a large diversity of contexts and backgrounds [50]. A CNN algorithm was trained to recognize IHs, with a final 91.7% diagnostic accuracy. The authors utilized ResNet [51], a standard open-source CNN architecture that has gained widespread use in the computer vision field for its impressive results across various domains, and applied 10-fold cross-validation to ensure the robustness of the results. The dataset size is significant, particularly in the pediatric dermatology context, as it encompasses images collected (and more importantly, labeled by a pediatrician) over a span of 17 years.

The original studies on the application of AI in pediatric dermatology are reported in Table 2.

The study by Mehta et al. [52], based on the diagnosis of pigmentary lesions and classification into benign or malignant lesions, confronted the efficiency of an algorithm set on the adult dermoscopic dataset before and after the addition of pediatric images. More precisely, the proposed approach exploits gradient-weighted class activation maps [53] and background skin masking to provide visual explanations that reveal the contributions of the image patches containing skin lesions and background patches. Two image classification models were then trained, one using images from a large dataset containing data on adult skin lesions and another trained with a combination of images from the first dataset plus pediatric skin lesion images. The latter’s inclusion improved the AI model’s performance on pediatric images while maintaining a high level of performance on adult images [52]. To expand these algorithms, it is advisable to collect non-standardized clinical images that differ in background, age, illumination, and appearance, as recommended by general studies for AI applied to computer vision and skin dermatology [49].

Another example of AI’s application in the field of rare pediatric dermatological diseases is the automatic recognition of the phenotype of X-linked hypohidrotic ectodermal dysplasia (XLHED) from facial images, as reported in a study by Hadj-Rabia et al. [54]. Using 157 images of patients plus controls during training, the underlying automated facial recognition technology was able to detect males with XLHED and recognize other forms of ectodermal dysplasia.

A study by Huang et al. [55] points out the potential of AI tools to support the decision-making process and medical knowledge of physicians, by testing ChatGPT-4.0 in multiple-choice and case-based questions, while also stressing the importance of improvement and medical supervision in the use of these technologies.

AI should not be seen as a replacement for a clinical encounter but rather as a support to strengthen patient care and raise the proficiencies of dermatologists.

## 4. Discussion

### 4.1. Challenges in Pediatric Dermatology Telemedicine and AI

As discussed in the previous sections, telemedicine and AI have emerged as revolutionary forces within the domain of pediatric dermatology, introducing remarkable capabilities in image recognition and data analysis. The incorporation of AI into the healthcare domain offers a multitude of benefits. Through the analysis of large volumes of data, AI can automate tasks such as detecting anomalies and finding the causes of and preventing disease. Overall, AI is capable of delivering faster, higher-quality healthcare services at a lower cost. AI could help tailor healthcare services to the individual patient.

However, the integration of AI into clinical practice faces a range of challenges encompassing technical, ethical, and regulatory aspects. This becomes especially relevant following the approval of the AI Act [56], which designates AI systems applied in the healthcare domain as high-risk. Consequently, it is imperative to ensure that the development, deployment, and utilization of AI systems align with the seven fundamental requirements for Trustworthy AI (TAI) [57]: (1) human agency and oversight; (2) technical robustness and safety; (3) privacy and data governance; (4) transparency; (5) diversity, non-discrimination, and fairness; (6) environmental and societal well-being; and (7) accountability.

The challenges and possible mitigation measures are discussed in detail below.

#### 4.1.1. Ethical and Regulatory Challenges

Several trustworthiness challenges must be carefully addressed to ensure the seven requirements for TAI are addressed, especially in the context of pediatric patients.

##### Lawfulness

Adherence to healthcare regulations, particularly the Health Insurance Portability and Accountability Act (HIPAA), is mandatory to safeguard patient rights and uphold the integrity of the healthcare system. In this regard, certifying bodies are needed to ensure compliance with regulations.

##### Human Agency and Oversight

This requirement involves retaining human control over critical decisions, ensuring transparency, and explaining AI outputs. The “human-in-the-loop” approach combines AI with human professionals to ensure balanced decision-making.

##### Technical Robustness and Safety

Due to the nature of the healthcare sector, AI solutions should be reliable in every circumstance. The design should be so robust that it can handle situations like missing or erroneous data, e.g., incorrect recording in patient information systems. At the same time, perfect accuracy or reliability cannot be obtained; hence, AI solutions should always be paired with human practitioners; they should be used to suggest rather than make decisions [55].

##### Privacy and Data Governance

A significant impediment to the use of SAF teledermatology resides in liability and medicolegal concerns and implications [58,59]. A study published in 2019 did not report cases of medical negligence in the field of direct-to-consumer telemedicine [60]. However, the fact that patient privacy could be compromised at various points in the image acquisition, transmission, and storage process remains a concern. Data privacy is especially crucial in the European Union, where data processing must comply with the General Data Protection Regulation (GRPR) [61], which has very stringent policies. Advancements in this field could alleviate such concerns and encourage dermatologists to extend their professional insights via SAF teledermatology [58]. Achieving this will necessitate a transformation in data security and privacy, along with a renewed focus on innovation through collaborative efforts within regulatory agencies and among software vendors [62].

Ensuring the confidentiality of sensitive patient information is a major concern in pediatric dermatology telemedicine.

Moreover, the choice of communication platforms plays a key role in protecting patient–doctor interactions. Secure telecommunication tools, armed with end-to-end encryption and secure video conferencing capabilities, serve as ramparts against privacy breaches during virtual consultations.

Securing informed consent from parents or legal guardians is a cornerstone of ethical pediatric dermatology telemedicine.

##### Transparency

Transparency in AI decision-making is key to fostering trust among healthcare providers and parents. AI algorithms should be designed to provide clear and understandable explanations for their diagnostic outputs.

##### Diversity, Non-Discrimination, and Fairness

Pediatric dermatology introduces unique challenges due to the diverse nature of skin conditions among children. A type of telemedicine tailored to various ethnic groups could overcome the obstacle posed by the fact that clinical manifestations of common pediatric skin conditions can vary significantly in individuals with darker skin tones. This highlights the need for access to experienced pediatric teledermatology platforms that can effectively accommodate patients from different ethnic backgrounds [44,63,64].

Dermatology AI algorithms perform significantly worse on lesions appearing on dark skin compared to light skin, resulting in a substantial decline in overall performance in clinical applications. In addition, it can deepen already existing discrepancies. To address this limitation, dermatology datasets used for AI training should include images of dark skin tones [65]. Diverse datasets, encompassing a representative range of pediatric cases, are essential to ensuring fairness and accuracy in assessments.

##### Environmental and Societal Well-Being

This involves designing AI systems that consider resource efficiency and waste reduction. Societal well-being emphasizes equity, inclusivity, and privacy protection, ensuring fair access, diverse representation, and robust data security.

#### 4.1.2. Technical Challenges

##### Interoperability

Another practical obstacle currently present in the use of teledermatology is the lack of interoperability between electronic medical records and many current teledermatology platforms. An application that integrates with existing electronic health records facilitates the review of medical history and past medications as well as communication between the dermatologist, the patient, and the primary care pediatrician [24]. Several healthcare facilities use diverse legacy systems that may not be easily compatible with AI technologies. Ensuring seamless integration with existing infrastructures is a significant challenge. The lack of standardized protocols for communication and interoperability between different AI systems and medical devices can hinder collaboration and data sharing.

##### Data Quality and Availability (or Scarcity)

An obstacle to implementing AI in pediatric dermatology arises from the predominance of studies focused on adult populations. This disparity results in a gap and inequality in the clinical utilization of this innovative tool, with applications for adults being more advanced than those for the pediatric population. Data available for the pediatric population are limited and of poor quality. To overcome this limitation, AI-based technologies need to be implemented based on datasets related to pediatric patients [49,64]. Furthermore, realistic synthetic data generation techniques specific to this scenario are needed. Attention must be given to training data biases when developing and deploying AI algorithms.

A technical limitation in the use of AI is related to the current scarcity of high-quality data, which requires the joint development of a package of programs. This limitation could be overcome by improvements in terms of enriching the functionality of the AI itself to improve its diagnostic accuracy [66] and through interdisciplinary collaboration between AI researchers and pediatricians. Moreover, the quality of the images transferred affects the outcome of the diagnosis; the use of standardized rules in the acquisition of photographs can improve AI’s performance [44,64,67]. Additionally, it should be noted that AI models are not immune to diagnostic errors. Therefore, clinical verification remains essential for all diagnoses. In this context, AI serves as a valuable tool for clinicians but cannot substitute them [49].

Data scarcity is an even bigger limiting factor when dealing with rare childhood diseases [49], as their low incidence is in itself a limitation to the dataset’s creation.

Another potential limitation of teledermatology is the limited ability to triage and obtain a comprehensive assessment of the patient due to the reliance on analyzing multiple individual photos, which cannot be a substitute for a total evaluation of the patient’s entire body [68,69]. The lack of specific questions or photo-related information worsens the efficiency of consultations [70]. Furthermore, teledermatology does not enable dermatologists to perform proper physical examinations, hence preventing the use of clinical clues obtained from palpation or second-level tests (e.g., swabs, biopsy, and videodermoscopy) [43,68].

Another potential technical limitation of telemedicine is that relying solely on images may result in the oversight of significant lesions and/or clinical clues located on the periphery of the images. The combined use of videos can overcome this limitation and better demonstrate the distribution, extent, and depth of skin lesions than photographs. Additionally, the recorded video can capture additional patient characteristics and behaviors. Therefore, in telemedicine, recorded videos in addition to still images may represent an effective way of transmitting more accurate information [42].

##### Model Flexibility and Specificity

Healthcare data are dynamic and continuously evolving. AI models need mechanisms to adapt to new information and updates in medical knowledge over time to adapt to new data and new knowledge. Moreover, AI models trained on specific datasets may lack the capacity to effectively extrapolate to heterogeneous patient populations or medical conditions. Ensuring the generalizability of models across various contexts is a significant challenge [71].

#### 4.1.3. Societal Challenges

##### Trust and Acceptance

The patient’s perception of this new tool can influence its use. According to Ukoha and colleagues [72], patients express concerns such as feeling neglected and less engaged with their healthcare providers, lacking the necessary equipment or training to monitor their health parameters at home, and perceiving virtual visits as a cost-cutting measure [72]. George et al. [73] examined perceptions about telemedicine among urban underserved minority populations in South Central Los Angeles. The study revealed that the African American population expressed more concerns about using telemedicine compared to the Latino population, particularly regarding privacy issues. This obstacle may be overcome by the personalized introduction of telemedicine to different ethnic groups, by raising awareness of the new tools available and providing education on their use.

Different cultural attitudes towards technology, data sharing, and AI can influence the acceptance and adoption of AI in healthcare. Understanding and addressing cultural variations is crucial. The implementation of AI in healthcare should enhance patient autonomy and streamline collaborative decision-making between healthcare providers and patients. Initiatives to educate the public about the benefits, risks, and ethical considerations of AI are essential for fostering acceptance.

Ultimately, the lack of clarity on the reimbursement system, understood as the payment received by a healthcare provider for providing a medical service, remains a significant barrier to the implementation and acceptance of teledermatology [4,58,59,74]. In 2021, the American Academy of Dermatology conducted an extensive survey involving 5000 participants, and the results of this survey highlighted that the majority of reported barriers were related to concerns about reimbursement [75]. The relatively low reimbursement rates for SAF teledermatology consultations may undervalue dermatologists’ time and expertise. This problem, highlighted by the U.S. healthcare system, can arise in any private healthcare system. Regularizing refund policies to make the teledermatology practice sustainable and ensuring reasonable reimbursement for teledermatology consultations could represent a solution to this problem.

##### Workforce Impact

Addressing the potential impact on employment and creating strategies for workforce transition are important social considerations. Healthcare professionals need adequate training to understand and effectively use AI technologies. It is also essential to ensure that medical education programs incorporate AI literacy.

##### Equity and Access

Teledermatology and telemedicine, in general, can overcome the barriers that limit access to medical care in underserved communities. The deployment of AI in healthcare should not exacerbate existing health disparities. There is a risk that certain populations may benefit more from AI applications, leading to inequities in healthcare access and outcomes. Ensuring that AI technologies are accessible to diverse populations, including those with limited financial resources or who live in remote areas, is crucial for equitable healthcare delivery.

Certain social determinants can impede the use of telemedicine. These include lacking ownership of electronic devices (such as smart devices or computers), lacking access to a reliable and strong internet connection, lacking a private location for visits, and lacking digital literacy, which refers to the inability to use electronic devices, connect to the internet, and complete trial video visits. Another limitation for the use of telemedicine is represented by the possible language barrier [4,72]. A recent retrospective study on 3027 patients analyzed the use of teledermatology in the pediatric setting during the COVID-19 pandemic. It was found that patients who had a primary language other than English were less likely to access pediatric dermatology care [45]. Another single-center cross-sectional study, conducted in Chicago during the COVID-19 pandemic, found that identifying as Black or African American, having a preferred non-English language, and having public insurance are independent factors associated with disparities in telemedicine use among pediatric dermatology patients [76]. All these obstacles can be overcome through strategies to encourage the adoption and optimization of teledermatology, in particular through specific patient education initiatives, less complicated patient registration requirements, and, if necessary, translation services [4].

**Figure 2:**
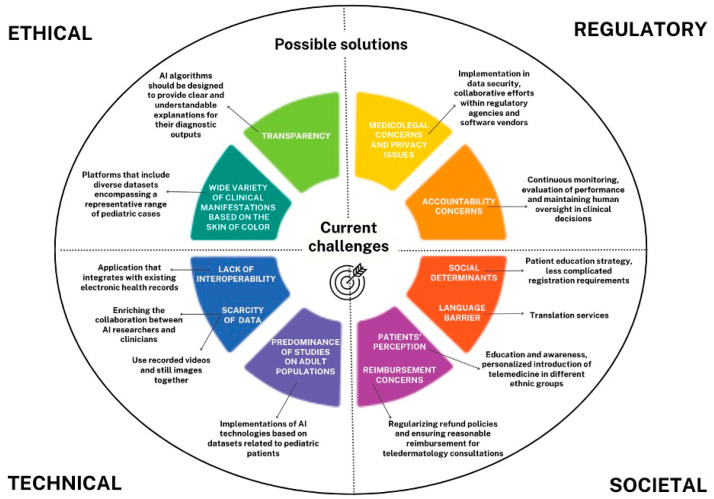
The current challenges and possible mitigation measures of telemedicine and AI in pediatric dermatology.

Figure 2 summarizes the current challenges and possible mitigation measures of telemedicine and AI in pediatric dermatology.

In this current scenario, the challenges present in AI approaches are diverse and include ethical and regulatory as well as technical and societal ones. The proper use of AI for social good entails that AI systems are not misused in critical contexts such as pediatric dermatology, i.e., without ensuring their adherence to the discussed requirements, thus making the system reliable. Therefore, it is necessary to find a way to ground these principles in practical techniques and tools that allow for the design and implementation of trustworthy systems to address the discussed challenges.

An example in this direction is the AEQUITAS (“Assessment and Engineering of Equitable, Unbiased, Impartial and Trustworthy AI Systems”) framework. The framework supports the development of an AI system throughout the entire pipeline. AEQUITAS allows for capturing the socio-technical context of the existing AI system to suggest quantitative fairness metrics. Controlled experiments can be constructed to compare different solutions, measuring and certifying the level of fairness for each. AEQUITAS enables the assessment of requirements for Trustworthy AI (TAI), evaluating their interplay and ensuring practical tools for directing the design and implementation of AI systems, especially in the medical field.

One case study of this framework focuses on pediatric dermatology. The scenario started with a dataset collected at Sant’Orsola University Hospital in Bologna, including various photos of children who accessed the pediatric emergency department for dermatological complaints, along with their medical history and diagnosis. The dataset initially faced the challenge of data scarcity for training an AI algorithm. The framework tackled this problem via the generation of realistic synthetic images using diffusion models, enabling the creation of a dataset large enough for training an AI algorithm. Within the framework, the dataset was analyzed to understand its content, potential imbalances, sensitive variables, and correlated variables. This helped to study AI fairness implications and enabled the application of the framework for designing and implementing a diagnostic support system in the field of pediatric dermatology, ensuring compliance with the fairness requirement. Algorithms can also be “stress-tested” within the framework to analyze possible breaking points (unfair behaviors) in the presence of highly imbalanced data. This “stress test” can be conducted within the framework by generating data with different levels of bias.

## 5. Conclusions

In this paper, we provide an overview of the state of the art in telemedicine and AI applications in pediatric dermatology, examining their current limitations and proposing potential solutions to address existing challenges. We review AI applications in pediatric dermatology, discussing their current capabilities, potential failure modes, and challenges related to performance assessment and interpretability (as well as more broadly, reliability). Our work presents some limitations such as the fact that the search is limited only to the last 15 years and includes only works written in English. However, this study can benefit the scientific community by organizing existing work on the topic and providing a detailed discussion of open challenges, with a particular focus on equity and ethical considerations associated with future clinical adoption. We concluded the survey by outlining possible concrete directions to foster trust in this process. The potential of AI in the field of pediatric dermatology can revolutionize patient care, particularly in improving the sensitivity and accuracy of screening of skin lesions, but specific research on the topic of providing trustworthy systems is necessary.

## Figures and Tables

**Table 1:** Original studies on the application of telemedicine in pediatric dermatology.

Author Year	Type of Study	Sample Size (Pediatric Population)	Age Range (Years)	Country	Primary Outcome	Secondary Outcomes	Significant Findings
Heffner et al., 2009 [16]	Cross-sectional study	135	3 months–18 years, 6 months	USA	To assess the ability of a pediatric dermatologist to accurately diagnose rashes based on history and digital images.	To determine interrater agreement on SAF images.	Concordance between in-person and photographic diagnosis by the primary dermatologist was 82%. Concordance between the two dermatologists on images was 73%, whereas interrater agreement between the two dermatologists, one viewing the patient in person and the other viewing photographs alone, was 69%.
Chen et al., 2009 [17]	Retrospective cohort study	429	0–12	USA	To identify the major problems emerging in the use of teledermatology in the pediatric population.		In total, 42% of diagnoses were discordant, especially for tinea versicolor, seborrheic dermatitis, pityriasis rosea, xerosis, and lichen striatus. Agreement in management between the PCP and teledermatologist was found in only 28% of cases. Topical steroids are underused by PCPs.
Tollefson et al., 2012 [20]	Cross-sectional study	30		USA	To examine early IH growth using parental photographs.	To assess the prevalence of hemangioma precursors evident at birth.	The majority of this growth occurs between 5.5 and 7.5 weeks of age. In total, 65% of patients had a hemangioma precursor on the first day of life.
Philp et al., 2013 [12]	Retrospective cohort study	395	0–18	USA	To assess whether historical data and the quality of photographs influence the ability to render a diagnosis.		A diagnosis was made in 75% of cases, regardless of the historical data provided. Previous treatments are the only data related to a more likely diagnosis. Poor image quality was not a significant barrier to providing a diagnosis.
Batalla et al., 2015 [13]	Retrospective and observational study	183	0–15	Spain	To describe the distribution of diseases consulted through teledermatology, avoiding FTF consultations, and the agreement between virtual and FTF diagnoses.		The most frequent diagnoses were inflammatory diseases (39%), benign pigmented lesions (23%), and infectious diseases (20%). In total, 48% of consultations were referred for a face-to-face diagnosis. The diagnostic agreement between the dermatologist who evaluated the virtual consultation and the one evaluating the FTF consultation was 89%.
Paradela et al., 2015 [32]	Retrospective and observational study	383	0–15	Spain	To evaluate the trustworthiness of SAF teledermatology and its potential to decrease FTF visits.		A total of 55.9% of diagnoses were concordant between pediatricians and teledermatologists. A lower accuracy was associated with incomplete clinical data or bad-quality photographic images. In total, 58.4% of FTF visits were avoided.
Feigenbaum et al., 2017 [42]	Controlled trial	31		USA	To assess whether recorded videos added to stationary images can enhance diagnosis and management in pediatric teledermatology, rather than relying solely on images.		Supplemental videos helped significantly with management accuracy. In less common conditions, the use of videos and still images outperformed the sole use of images in terms of management.
O’Connor et al., 2017 [14]	Prospective cohort study	40	0–18	USA	To evaluate concordance between diagnoses based on in-person examination and those based on parental photographs.	To assess the effect of photography instructions on in-person photograph-based vs. examination-based diagnoses.	Overall concordance between photograph-based and in-person diagnoses was 83%. The provision of photographic instruction did not statistically influence diagnostic concordance.
Bridges et al., 2019 [40]	Retrospective cross-sectional study	44		USA	To assess the effect of Dermatology ECHO, a telemonitoring program connecting PCPs and specialists, over 2 years.		In total, 45% of patients had correct diagnoses. Among them, 77% profited from expert treatment recommendations.
Bergamo et al., 2020 [43]	Monocentric prospective study	32		Italy	During the COVID-19 pandemic, a direct line for teledermatology was provided, where a dermatologist replied to all GP and pediatrician requests.		In 86% of teledermatology consultations, diagnosis and management were provided without the need for a visit. Teledermatology does not allow for second-level investigations during the visit. This can cause a delay in diagnosis.
Marchetti et al., 2020 [25]	Retrospective cohort study	290 (44 children)	0–15	France	To assess diagnostic concordance in tertiary (dermatologist-to-expert) teledermoscopy and its efficiency.		Diagnostic concordance was found in 77% of cases. Final concordance on the benign or malignant nature of the lesion was observed in 77.3% of cases.
Betlloch-Mas et al., 2020 [21]	Retrospective descriptive study	432	0–14	Spain	To evaluate the role of teledermatology as a tool in pediatric settings.	To assess whether teledermatology was effective in reducing the age of propranolol initiation in presumed IH.	In total, 48.12% of cases consulted via teledermatology were resolved remotely. After the implementation of telemedicine (2015–2018), children with IHs began treatment at a mean age of 4.5 months, before which treatment began at 7.1 months (2008–2014).
Seiger et al., 2020 [29]	Retrospective cohort study	188	0–18	USA	To assess the duration of waiting times and the avoidance of FTF dermatology visits via a pediatric dermatology eConsult program.	To assess recommendations for FTF dermatology visits and potential cost savings.	In total, 31.9% of cases were referred to FTF evaluation. After the eConsult, the mean wait time for an initial FTF evaluation was reduced by 31%. The program acted as a triage to avoid FTF visits and offered cost savings.
Jew et al., 2020 [31]	Prospective non-blinded cohort study	43	0–18	USA	To evaluate the efficiency of a provider-to-provider SAF teledermatology consultation process.	To assess the acceptance of SAF teledermatology among patients/parents, PCPs, and dermatologists.	The median time for PCPs to communicate teledermatology recommendations to families was 3 days. In-person follow-up visits after telemedicine were required for 23%. A total of 83% of parents, as well as all PCPs and dermatologists, were satisfied with the service.
Calafiore et al., 2021 [30]	Retrospective cohort study	876		USA	To assess the effect of a SAF teledermatology program augmented by the incorporation of dermoscopy in pediatric patient health centers.		All 536 telemedicine referrals received dermatological care within 24 h, whereas with the traditional system, the mean time to be visited by a dermatologist was 75 days. Only 12% of referrals were recommended for follow-up visits.
Pahalyants et al., 2021 [15]	Retrospective study	310	0–22	USA	To identify patients and variables associated with an efficient diagnosis and management in the SAF service.	To assess pediatrician and parental openness to teleconsultations.	Follow-up visits were recommended in 28% of cases. There was full concordance in the diagnoses of 70.1% of patients seen subsequently. A survey revealed that teleconsultations were received positively by pediatricians and parents.
Cline et al., 2022 [37]	Multicenter retrospective analysis	3659		USA	To evaluate the effect of telemedicine on no-show rates in pediatric dermatology from 3 safety-net clinics.		Telemedicine was linked to a significantly lower non-attendance rate at each site.
Kohn et al., 2022 [38]	Prospective single-center study	519		USA	To assess the pertinency of synchronous (live video) teledermatology in a pediatric setting.		Patient satisfaction (84.3%) with telehealth consultations was more likely to be higher compared to dermatologists (68.4%). A photo to support dermatologists in their examination was reported in 10.7% of cases.
Lowe et al., 2022 [18]	Prospective single-center cohort study	116	1 month–17 years	Wales	To measure the potential of a virtual pediatric dermatology telephone clinic, both from the clinician and patient/parental perspective.		From the clinician’s viewpoint, most consultations (91%) were successfully concluded over the telephone. In total, 52% of parents felt unsatisfied as the majority (65%) preferred FTF follow-ups in the future.
Kittler et al., 2022 [23]	Multicenter cross-sectional study	281		USA	To appraise the experiences of hemangioma specialists in managing IH using telemedicine.		The median time from referral to evaluation was 17 days. Median physician confidence in performing telemedicine evaluations was 95.0. Hybrid telemedicine and the review of photographs were favored as modalities.
Duan et al., 2022 [5]	Single-center cross-sectional study	1444	0–18	USA	To identify factors associated with disparities in telemedicine use among the pediatric dermatology population during the COVID-19 pandemic.		Being Black or African American, having a preferred non-English language, and not having public insurance represented factors of reduced use of telemedicine.
Hansen et al., 2023 [35]	Retrospective cross-sectional study	504	1 month–17.8 years	Germany	To describe the dermatological requests submitted by pediatricians to a dermatologist using SAF technology.		A definite diagnosis was made in 88.3% of cases. In total, 90% of the requests were processed on the same or following day.
Ragamin et al., 2023 [44]	Prospective observational study	87	4–12	The Netherlands	To assess the validity of the Eczema Area and Severity Index (EASI) based on images and patient-assessed severity based on the Self-Administered EASI (SA-EASI).		Excellent validity (0.90), good inter- (0.77) and intrarater reliability (0.91), and standard error of measurement (4.31) were found for the EASI based on clinical images. The quality of images influences the assessment. A moderate correlation was found (0.60) between SA-EASI and EASI.
Zacher et al., 2023 [45]	Cross-sectional retrospective study	3027	<18	USA	To examine whether variables such as geographic residence, ADI, ethnicity, race, or insurance type influence the use of teledermatology in pediatric settings.		Patients with a primary language other than English were less likely to access pediatric dermatology care. No significant differences were found in those variables between patients seen only in person and those seen only through telehealth.
Taslidere et al., 2023 [19]	Prospective observational study	93	0–16		To determine the group of pediatric patients in which teledermatology has the highest success rate in diagnosing dermatological lesions.		Diagnostic concordance between face-to-face and teledermatology diagnoses was 74%. The agreement rate was variable between different lesions: 100% for acne and scabies, and 25% for contact dermatitis.
Ying et al., 2024 [26]	Retrospective review	162		New Zealand	To analyze how specialist advice influences GPs’ prescribing practices and the impact of teledermatology in the management of pediatric eczema patients.		Following a dermatology specialist’s advice, an important change in the prescribing patterns of medications was observed. Even if it is effective, teledermatology is insufficient to guarantee the correct adoption of pediatric eczema guidelines by GPs.

GP: general pediatrician; IH: infantile hemangiomas; ECHO: Extension for Community Healthcare Outcomes; PCPs: primary care providers; SAF: store and forward; FTF: face to face; ADI: Area Deprivation Index.

**Table 2:** Original studies on the application of AI in pediatric dermatology.

Author Year	Type of Study	Sample Size (Pediatric Population)	Age Range (Years)	Country	Primary Outcome	Secondary Outcomes	Significant Findings
Zhang et al., 2022 [50]	Training and validation study	5834	0–1	USA	An AI algorithm was trained to recognize infantile hemangiomas based on clinical images.		The algorithm reached 91.7% accuracy in the diagnosis of facial IH.
Mehta et al., 2023 [52]	Training and validation study	1536	0–18	Australia	To compare the rendering of an AI model trained on a standard adult-predominant dermoscopic dataset before and after the addition of pediatric images.		Pediatric images enhanced the algorithm’s efficiency, maintaining high performance on adult images.

AI: artificial intelligence; IH: infantile hemangiomas.

## Data Availability

No new data were created in this study. Data sharing is not applicable to this article.
